# Individual and community-level determinants of pentavalent vaccination dropouts among under-five children in the sub-Saharan African countries: A multilevel analysis of the recent demographic and health survey

**DOI:** 10.1016/j.jvacx.2024.100465

**Published:** 2024-02-22

**Authors:** Alebachew Ferede Zegeye, Chilot Kassa Mekonnen, Hailemichael Kindie, Belayneh Shetie Workneh, Desale Bihonegn Asmamaw, Tadesse Tarik Tamir

**Affiliations:** a Department of Medical Nursing, School of Nursing, College of Medicine and Health Sciences University of Gondar, Gondar, Ethiopia; b Department of Emergency and Critical Care Nursing, School of Nursing, College of Medicine and Health Sciences, University of Gondar, Gondar, Ethiopia; c Department of Reproductive Health, Institute of Public Health, College of Medicine and Health Sciences, University of Gondar, Gondar, Ethiopia; d Department of Pediatrics and Child Health Nursing, School of Nursing, College of Medicine and Health Sciences, University of Gondar, Gondar, Ethiopia

**Keywords:** Determinants, Dropout, Pentavalent vaccination, Prevalence, Sub-Saharan Africa

## Abstract

**Background:**

Globally, each year, about 3 million deaths among children are prevented by pentavalent vaccinations. However, in developing countries, particularly in Sub-Saharan Africa, pentavalent vaccination dropout rates are not well reported. Therefore, this study aimed to assess pentavalent vaccination dropout rates and their determinants among under-five children in Sub-Saharan Africa.

**Methods:**

Data from the recent Demographic and Health Surveys in 33 Sub-Saharan African countries were used for analysis. The study used a total of 358,529 under-five children. The determinants of pentavalent vaccination dropout were determined using a multilevel mixed-effects logistic regression model. Significant factors associated with pentavalent vaccination dropout were declared significant at p-values < 0.05. A model with the lowest deviance and highest logliklihood ratio was selected as the best-fit model.

**Results:**

In Sub-Saharan Africa, one in five under-five children had pentavalent vaccination dropout rates. Factors such as Age (AOR = 1.61, 95 % CI: 1.51, 1.72), educational level (AOR = 1.30, 95 % CI: 1.22, 1.40), place of delivery (AOR = 1.65, 95 % CI: 1.57, 1.73), ANC visits (AOR = 1.34, 95 % CI: 1.23, 1.45), postnatal check-up (AOR = 1.19, 95 % CI: 1.14, 1.25), wealth status (AOR = 1.09, 95 % CI: 1.04, 1.15), distance to health facility (AOR = 1.08, 95 % CI: 1.03, 1.13), media exposure (AOR = 1.12, 95 % 1.15), and geographical region (AOR = 1.60, 95 % CI: 1.49, 1.72) had higher odds of pentavalent vaccination dropouts.

**Conclusions:**

Pentavalent vaccination dropout rates in sub-Saharan Africa among under-five children were high. Both individual and community-level variables were determinants of pentavalent vaccination dropout rates. Government and ministry of health in Sub-Saharan Africa should give attention to those mothers of under-five children who reported distance as a big problem in accessing health facilities and to women who do not utilise antenatal and postnatal check-ups while designing policies and strategies in sub-Saharan Africa.

## Background

Immunization is the process of using vaccines to protect the body from an infectious disease attack [1]. Since vaccination ensures protection against major childhood diseases and thereby prevents millions of deaths and cases of disability globally, childhood immunization is a major significant indicator of good child health [2]. One of the most effective health initiatives available to reduce the mortality and morbidity of infectious diseases in children is childhood immunization, which prevents 1–2 million childhood deaths globally [3].

Since the Expanded Programme on Immunization was introduced, there have been significantly fewer deaths among under-five children throughout the world, from 12.5 million in 1990 to 5.2 million in 2020 [4]. Globally, each year, about 3 million deaths among children are prevented from vaccine-preventable diseases such as diphtheria, tetanus, pertussis, influenza, and measles. On the other hand, each year, vaccine-preventable diseases cost the lives of 8.8 million children under the age of five. Sub-Saharan Africa and Central and Southern Asia account for about 80 % of the world's child deaths because of incomplete vaccination coverage [5], [6], [7].

Although there has been a dramatic decrease in the incidence of vaccine-preventable mortality, a substantial number of children are not fully vaccinated, leading to significant regional and global variation in vaccination coverage [8], [9]. For instance, 19.7 million children globally did not receive the third dose of the diphtheria, tetanus, and pertussis (DTP3) vaccination during their first year of life in 2019, which is an essential indicator of the effectiveness of immunization efforts [10], [11]. In sub-Saharan Africa, 4.4 million children lose their lives due to communicable diseases each year as a result of inadequate vaccination and setup, incomplete vaccination, and impediments to delivering vaccinations, although these deaths may have been avoided by vaccination [2], [12], [13].

According to the WHO's global strategy for the Immunization Agenda 2030, regardless of their geographic location, age, financial situation, or gender-related limitations, every child should have received all recommended immunizations by the year 2030. This agenda focuses on the eight vaccine-preventable childhood diseases like tuberculosis, diphtheria, pertussis (whooping cough), tetanus, polio, measles, hepatitis B, and Haemophilus influenza type B [Hib) [14], [15].

A pentavalent vaccination dropout occurs when a child has received the first recommended dose of the vaccine but has missed the third recommended pentavalent vaccination dose. Vaccination dropout is used as an indicator of immunization program performance and low dropout rates indicate good access and utilization of immunization services [16]. DTP1 to DTP3, BCG to measles-containing virus (MCV1), and MCV1 to MCV2 were suggested by the World Health Organization (WHO) as indications of immunization dropout. According to the WHO, if the dropout rate is greater than 10 %, it means that many people fail to use the services [16], [17].

The studies carried out in different parts of the world documented that different determinants were significantly associated with vaccination dropouts among children. Postnatal care, and having a mother who did not receive tetanus toxoid (TT) vaccination [18], mothers with less than 4 or no antenatal care (ANC) visits, long distances to the health facilities, and mothers without formal employment [19], having a mother with below secondary education [20], children who had no immunization cards [21], and residence [22] were found to be significant predictors of vaccination dropouts among children.

Despite achievements observed in the reduction of under-five mortality rates, about 1–2 million children are still dying each year due to vaccine-preventable diseases, and sub-Saharan African countries share the huge burden of global under-five mortality. As far as our search of the literature and knowledge goes, there have been no studies conducted using DHS data on pentavalent vaccination dropout rates and their determinants, especially community-level determinants, in sub-Saharan African countries.

Therefore, the present study focuses on investigating the individual and community-level determinants of pentavalent vaccination dropouts among under-five children in sub-Saharan African countries using a multilevel mixed effect analysis of the recent Demographic and Health Survey data. Furthermore, the current study's findings offer support for health planners, policymakers, sponsors, and health professionals as they desire to further reduce pentavalent vaccination dropouts among under-five children, which would assist in lowering under-five mortality due to vaccine-preventable diseases in countries with middle and low incomes, such as sub-Saharan Africa.

## Methods

### Study setting

The sub-Saharan is the area of the continent of Africa that lies south of the Sahara and consists of four vast and distinct regions, such as Eastern Africa, Central Africa, Western Africa, and Southern Africa. Together, they constitute an area of 9.4 million square miles and a total population of 1.3 billion inhabitants. According to the UNICEF estimate, by 2055 there will be one billion under-5 children [23], [24]. This study was conducted based on the recent DHS survey data from thirty-three sub-Saharan African countries such as Angola, Burkina Faso, Benin, Burundi, DR. Congo, Congo, Ivory coast, Cameron, Ethiopia, Gabon, Ghana, Gambia, Guinea, Kenya, Comoros, Liberia, Lesotho, Mali, Malawi, Mozambique, Nigeria, Niger, Namibia, Rwanda, Serra Leone, Senegal, Chad, Togo, Tanzania, Uganda, South Africa, Zambia, and Zimbabwe.

### Study design and period

A community-based multilevel mixed-effect cross-sectional study was conducted. A recent DHS survey of data from 33 sub-Saharan African countries, which was conducted between 2010 and 2020, was used to carry out a multilevel mixed effect analysis. The Demographic and Health Survey (DHS) is a national-level study conducted every five years using structured, pretested, and validated tools as part of the worldwide Demographic and Health Survey. To get a representative sample of recent Demographic and Health Survey data from each region of sub-Saharan African countries, 10 years of DHS data (starting from 2010) were taken. The surveys have huge sample sizes, are population-based, and nationally representative of all countries.

### Population and eligibility criteria

Under-five children who are 0–59 months old in Sub-Saharan African countries were the source population. The study population was all the under-five children who were in the selected enumeration areas included in the analysis.

### Data source and sampling procedure

The DHS survey data from 33 sub-Saharan countries was appended together to investigate pentavalent vaccination dropout and determinates among under-five children. The survey for every country contains different datasets; including data on basic health indicators like mortality, morbidity, family planning service utilization, fertility, maternal and child health services such as vaccination. The Demographic and Health Survey has a stratified two-stage cluster design that, in the first stage, comprises the enumeration areas and, in the second stage, generates a sample of households from each enumeration area. For this study, we used the Kids record dataset (KR file) to extract the dependent and independent variables for each country and then we append the data using the STATA. The variables “received pentavalent-1(h51) and pentavalent-3 (h53)” from the kids record (KR) data set was recoded to create the outcome variable (pentavalent vaccination dropout). The variables that are associated with pentavalent vaccination dropout rates were identified using a binary logistic regression model. Determinants of the pentavalent vaccination dropout were reported in terms of an adjusted odds ratio (AOR) with a significance level of (95 %). In the univariate analysis, at 95 % confidence intervals with a p-value of < 0.25 was considered a candidate for the multivariable analysis of data. All variables with p values < 0.05 were considered statistically significant in multivariable logistic regression. The study comprised a weighted sample of 358,529 children under the age of five (Table 1).

**Table 1:** Sample size for Individual and community-level determinants of pentavalent vaccination dropouts among under-five children in the Sub-Saharan African countries, DHS 2010–2020.

Country	Year of survey	Weighted sample (n)	Weighted sample (%)
Angola	2015/16	14,322	3.99
Burkina Faso	2010	15,044	4.2
Benin	2017/18	13,589	3.79
Burundi	2016/17	13,192	3.68
DR. Congo	2013/14	18,716	5.22
Congo	2011/12	9,329	2.6
Ivory coast	2011/12	7,776	2.17
Cameron	2018	9,733	2.71
Ethiopia	2016	10,641	2.97
Gabon	2012	6,067	1.69
Ghana	2014	5,884	1.64
Gambia	2019/20	8,362	2.33
Guinea	2018	7,951	2.22
Kenya	2014	20,964	5.85
Comoros	2012	3,149	0.88
Liberia	2019/20	5,704	1.59
Lesotho	2014	3,138	0.88
Mali	2018	9,940	2.77
Malawi	2015/16	17,286	4.82
Mozambique	2011	11,102	3.1
Nigeria	2018	33,924	9.46
Niger	2012	12,558	3.5
Namibia	2013	5,046	1.41
Rwanda	2019/20	8,092	2.26
Serra Leone	2019	9,899	2.76
Senegal	2019	6,125	1.71
Chad	2014/15	18,623	5.19
Togo	2013/14	6,979	1.95
Tanzania	2015/16	10,233	2.85
Uganda	2016	15,522	4.33
South Africa	2016	3,548	0.99
Zambia	2018	9,959	2.78
Zimbabwe	2015	6,132	1.71
Total Weighted sample size		358,529	100

### Study variables

**Dependent variables**: The outcome of this study was pentavalent vaccination dropout, in which the child who received the first antigen of the pentavalent but not the third antigen of the pentavalent, or the first antigen of the pentavalent but not the first antigen of measles [19]. The pentavalent vaccination dropout was assessed by recoding the variables pentavalent 1 (h51) and pentavalent 3 (h53) from the Kids Record (KR) data set. The pentavalent vaccination dropout rate was calculated by dividing the number of children aged 0–59 months who received pentavalent 1 by the number of children aged 0–59 months who received pentavalent 3 divided by the number of children 0–59 months of age who received Pentavalent 1 multiplied by 100 %; $\frac{Pentavalent1-Pentavalent3}{Pentavalent1}\times 100\%$. The pentavalent dropout rate can also be calculated by using the percentage of children aged 0–59 months who received pentavalent 1 minus the percentage of children aged 0–59 months who received measles 1, divided by those who received pentavalent 1 multiplied by 100 % $\frac{Pentavalent1-measlesvaccine}{Pentavalent1}\times 100\%$ (17).

**Independent variables**: Independent variables from two sources (variables at the individual and community levels) were taken into account for this analysis because DHS data are hierarchical. The independent variables at the individual level were: Sex of child (Male, Female), Sex of household head (Male, Female), Birth interval (months) (<24, 24–28, >48), Maternal age [15–24, 25–34, 35–49), Maternal educational status (No formal education, Primary, Secondary and higher), Husband educational status (No formal education, Primary, Secondary and higher), Maternal employment/occupation (No, Yes), Marital status of the mother (Unmarried, Married, Ever married), Place of delivery (Home, Health facility), Mode of delivery (vaginal delivery, caesarean section delivery), Wanted pregnancy (Yes, No) Number of ANC visits (<4, ≥4), Postnatal checkup within 2 months (No, Yes) Presence of immunization card (No, Yes, Unknown) TT vaccine during pregnancy (No, Yes), Number of under-5 children (≤1, 2, ≥3), Household wealth index (Poor, Middle, Rich), Birth order number), 1st order, 2–4, >4), Distance to health facility (Big problem, Not big problem), and Household media exposure (No, Yes). The community-level variables were Place of residence (Urban, Rural), Community level women illiteracy (Low, High), Community level poverty (Low, High), Community level media exposure (Low, High), Community level ANC utilization (Low, High), and Country category (West SSA, East SSA, Central SSA, South SSA).

### Data processing and statistical analysis

The data were extracted from recent DHS data sets and cleaned, recorded, and analyzed with STATA version 14 Statistical Software. Before conducting any statistical analysis, the data were weighted using the sampling weight, primary sampling unit, and strata in order to restore the survey's representativeness and account for the sampling design when computing standard errors to generate accurate statistical estimations. We used the weighting variable (v005) as a relative weight normalized to make the analysis survey-specific, while for the pooled data, we denormalized the under-five children’s individual standard weight variable by dividing the under-five children’s individual standard weight by the sampling fraction of each country: (under-five children adjusted weight = V005× (total under-five children aged 0–59 years in the country at the time of the survey)/ (number of under-five children aged 0–59 years in the survey).

The assumptions of standard logistic regression model such as independence observations and equal variance are broken due to the hierarchical nature of the DHS data. for instance, mothers and children are nested within a cluster, and we assume that the subjects in one cluster may have similar characteristics to those in another, which goes against the equal variance and independence assumptions between clusters in the ordinal logistic regression model. This implies that accounting for between-cluster effects requires the use of an advanced model. Given this, multilevel mixed-effects logistic regression was used to determine the factors that associated with pentavalent vaccination dropout. Four models are used in multilevel mixed effect logistic regression: model I (only individual level variables), model II (only community level variables), model III (both individual and community level variables), and the null model (outcome variable only). The model without independent variables (null model) was used to check the variability of pentavalent vaccination dropout rates across the cluster. It was identified which community-level variables and which individual-level variables were associated with the outcome variable (Model II) and Model I, respectively. In the final model (Model III), the association of both individual and community-level variables was fitted simultaneously with the outcome variable (pentavalent vaccination dropout rate).

### Random effects (Measures of variation)

Random effects or measures of variation such as Likelihood Ratio test (LR), Intra-class Correlation Coefficient (ICC), and Median Odds Ratio (MOR) were computed to measure the variation of pentavalent vaccination dropout rates across clusters. Taking clusters as a random variable, the ICC quantifies the degree of heterogeneity of pentavalent vaccination dropout rates between clusters (the proportion of the total observed variation in pentavalent dropout that is attributable to between cluster variations [25] is computed as; ICC=$\frac{\mathrm{VC}}{\mathrm{VC}+3.29}\times 100\%$. The Median Odds Ratio (MOR) is the median value of the odds ratio which quantifies the variation or heterogeneity in pentavalent vaccination dropout rates between clusters in terms of odds ratio scale and is defined as the median value of the odds ratio between the cluster at high likelihood of pentavalent vaccination dropout rates and cluster at lower risk when randomly picking out individuals from two clusters[26]; MOR= e ^0.95√VC^.

Moreover, the PCV demonstrates the variation in the pentavalent vaccination dropout rates explained by determinants and computed as; PCV=$\frac{\mathrm{Vnull}-Vc}{\mathrm{Vnull}}\times 100\%$; where Vnull = variance of the null model and VC = cluster level variance [27]. The fixed effects were used to estimate the association between the likelihood of pentavalent vaccination dropout rates and individual and community level independent variables. It was assessed and the strength was presented using adjusted odds ratio (AOR) and 95 % confidence intervals with a p-value of < 0.05. Because of the nested nature of the model, Deviance = -2(log likelihood ratio) was used to compare models, and the model with the lowest deviance and the highest log likelihood ratio was selected as the best-fit model. The variables used in the models were verified for multi-collinearity by measuring the variance inflation factors (VIF), with the findings falling within acceptable limits of one to ten.

## Result

### Sociodemographic and economic characteristics of under-five children in sub-Saharan Africa countries

A total of 358,529 under-five children (181,344 males and 177,185 females) were included in the analysis. Nearly one-third 107,708 (30.04 %) of the under-five children were born to mothers with no occupation and 119,409 (40.48 %) were born to mothers with no formal education. about two-third 249,967 (69.72 %) of the under-five was born to mothers living in rural areas of sub-Saharan Africa, and more than half (58.3 %) of the children were born to mothers living in a community with a low level of literacy (Table 2).

**Table 2:** Sociodemographic and economic characteristics of under-five children in sub-Saharan Africa countries.

**Individual level variables**	Category	Frequency (n)	Percent (%)
Sex of child	Male	181,344	50.58
	Female	177,185	49.42
Sex of household head	Male	284,397	79.32
	Female	74,132	20.68
Birth interval (months)	< 24	55,002	19.60
	24–28	160,298	57.14
	> 48	65,257	23.26
Maternal age	15–24	102,670	28.64
	25–34	171,520	47.84
	35–49	84,339	23.52
Maternal educational status	No formal education	145,143	40.48
	Primary	119,409	33.31
	Secondary and higher	93,977	26.21
Husband educational status	No formal education	122,777	39.47
	Primary	84,844	27.27
	Secondary and higher	103,479	33.26
Maternal employment/occupation	No	107,708	30.04
	Yes	250,821	69.96
Marital status of the mother	Unmarried	22,769	6.35
	Married	253,312	70.65
	Ever married	82,448	23.00
Place of delivery	Home	121,012	33.75
	Health facility	237,517	66.25
Mode of delivery	vaginal delivery	340,641	95.19
	caesarean section delivery	17,220	4.81
Wanted pregnancy	Yes	328,551	94.62
	No	18,693	5.38
Number of ANC visits	< 4	30,817	8.60
	≥ 4	327,712	91.40
Postnatal checkup within 2 months	No	136,856	59.85
	Yes	91,811	40.15
Presence of immunization card	No	7,320	2.04
	Yes	5,993	1.67
	Unknown	345,216	96.29
TT vaccine during pregnancy	No	63,246	17.64
	Yes	295,283	82.36
Number of under-5 children	≤ 1	122,142	34.07
	2	141,069	39.35
	≥ 3	95,318	26.59
Household wealth index	Poor	97,961	45.74
	Middle	42,186	19.70
	Rich	74,007	34.56
Birth order number	1st order	77,132	21.51
	2–4	171,135	47.73
	> 4	110,262	30.75
Distance to health facility	Big problem	140,286	42.05
	Not big problem	193,365	57.95
Household media exposure	No	135,368	37.82
	Yes	222,572	62.18
**Community level variables**			
Place of residence	Urban	108,562	30.28
	Rural	249,967	69.72
Community level women illiteracy	Low	209,016	58.30
	High	149,513	41.70
Community level poverty	Low	186,010	52.32
	High	169,515	47.68
Community level media exposure	Low	190,188	53.05
	High	168,341	46.95
Community level ANC utilization	Low	160,425	44.75
	High	198,104	55.25
Country category	West SSA	143,735	40.09
	East SSA	108,986	30.40
	Central SSA	76,790	21.42
	South SSA	29,018	8.09

### Prevalence of pentavalent vaccination dropout among under-five children in sub-Saharan African countries

**Fig. 1:**
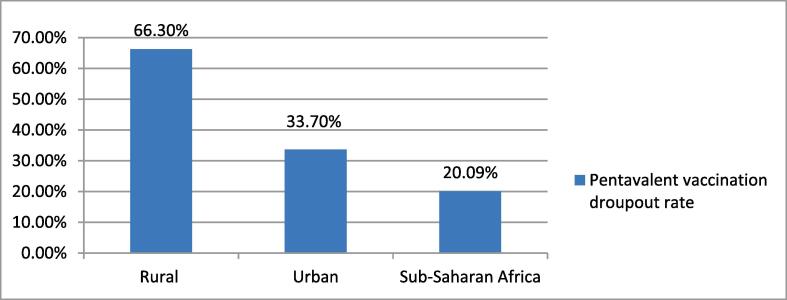
Pentavalent vaccination droupout rates among under-five children in the sub-Saharan African countries.

**Fig. 2:**
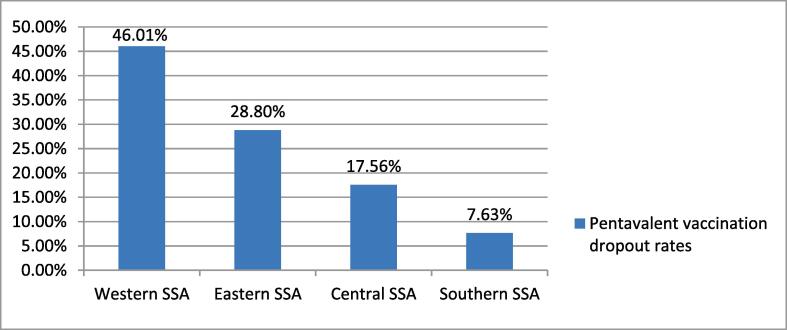
Regional prevalence of pentavalent vaccination dropout rates among under-five children in the sub-Saharan African countries.

The overall pentavalent vaccination dropout rate in the Sub-Saharan African countries was 20.9 % (95 % CI: 20.64, 21.16). The urban and rural pentavalent vaccination dropout rates in sub-Saharan African countries were found to be 33.7 % and 66.3 %, respectively (Fig. 1). The pentavalent vaccination dropouts among under-five children were significantly varied across the sub-Saharan African countries. Subsequently, the lowest pentavalent vaccination dropout was observed in the southern sub-Saharan African countries (7.63 %), while the highest was seen in the western sub-Saharan African countries (46.01 %) (Fig. 2).

### Random effect (Measures of variation) and model fitness

Findings from the null model showed that there were significant differences in pentavalent vaccination dropout between communities, with a variance of 0.0402511. In the null model, about 1.21 % of the total variation on pentavalent vaccination dropout was occurred at the cluster level and is attributable to the community-level factors. Furthermore, the null model exhibited the highest median odds ratio (MOR) value (1.21). This means that, when choosing an individual at random from one cluster at a higher risk of pentavalent vaccination dropout and another cluster at a lower risk, those in the higher risk cluster had 1.21 times higher odds of being pentavalent vaccination dropouts than those in the lower risk cluster. The intraclass correlation value for Model I indicated that 0.86 % of the variation in pentavalent vaccination dropout accounts for the disparities between communities. Next, using the null model, we developed Model II using variables at the community level. Cluster variations were the basis for 1.20 % of the differences in pentavalent vaccination dropout rate, according to the ICC value from Model II. In the final model (model III), which attributed approximately 8.19 % of the variation in the likelihood of pentavalent vaccination dropout rate to both individual and community-level factors. The model fitness was conducted by using logliklihood ratio and deviance in the final model (model 3), which was the best-fitted model since it had the lowest deviance (58,009.91) and the highest logliklihood ratio (-29004.955) (Table 3).(See Table 4).

**Table 3:** Model comparison and random effect analysis for pentavalent vaccination dropout rates among under-five children in the Sub-Saharan Africa countries.

Parameter	Null model	Model I	Model II	Model III
Community level variance	0.0402511	0.0284928	0.0397817	0.0369543
ICC	1.21 %	0.86 %	1.20 %	1.11 %
MOR	1.21	1.17	1.14	1.20
PCV	Reference	29.21 %	1.17 %	8.19 %
**Model fitness**				
LLR	−49179.559	−29375.245	−47878.744	−29004.955
Deviance	98,359.118	58,750.49	95,757.488	58,009.91

ICC: interacluster correlation, LLR: logliklihood ratio, MOR: median odds ratio, PCV: proportional change in variance.

**Table 4:** Multivariable multilevel logistic regression analysis of individual-level and community level factors associated pentavalent vaccination dropout rates among under-five children in Sub-Saharan Africa, DHS 2010–2020.

**Individual level factors**	Category	Model I AOR(95 % CI)	Model II AOR(95 % CI)	Model III AOR(95 % CI)
Sex of child	Male	0.99(0.95, 1.03)		0.99(0.95, 1.03)
	Female	1		1
Sex of household head	Male	1		1
	Female	1.08(1.02, 1.14)		1.06(0.99, 1.12)
Birth interval (months)	< 24	1		1
	24–28	0.94(0.89, 0.99)		0.96(0.91, 1.02)
	> 48	1.09(1.02, 1.17)		1.03(0.05, 1.21)
Maternal age	15–24	1.60(1.50, 1.70)		**1.61(1.51, 1.72)**
	25–34	1.19(0.13, 1.25)		1.14(0.11, 1.26)
	35–49	1		1
Maternal educational status	No formal education	1.31(1.23, 1.40)		**1.30(1.22, 1.40)**
	Primary	1.01(0.95, 1.08)		1.13(0.06, 1.20)
	Secondary and higher	1		1
Husband educational status	No formal education	0.92(0.87, 1.98)		0.72(0.86, 1.58)
	Primary	0.82(0.77, 1.86)		0.63(0.48, 1.99)
	Secondary and higher	1		1
Maternal employment/occupation	No	1.14(0.10, 1.19)		1.12(0.13, 1.24)
	Yes	1		1
Place of delivery	Home	1.76(1.67, 1.84)		**1.65(1.57, 1.73)**
	Health facility	1		1
Mode of delivery	vaginal delivery	1		1.08(0.98, 1.20)
Wanted pregnancy	Yes	1		1
	No	1.11(1.02, 1.20)		0.20(0.11, 1.31)
Number of ANC visits	< 4	1.34(1.24, 1.45)		**1.34(1.23, 1.45)**
	≥ 4	1		1
Postnatal checkup within 2 months	No	1.25(1.20, 1.31)		**1.19(1.14, 1.25)**
	Yes	1		1
Presence of immunization card	No	0.59(0.52, 1.66)		0.49(0.41, 1.38)
	Yes	0.63(0.57, 0.69)		0.51(0.44, 0.52)
	Unknown	1		1
TT vaccine during pregnancy	No	1.36(0.29, 1.43)		1.32(0.25, 1.39)
	Yes	1		1
Number of under-5 children	≤ 1	0.60(0.56, 1.64)		0.55(0.41, 0.60)
	2	0.75(0.71, 0.79)		0.71(0.67, 0.75)
	≥ 3	1		1
Household wealth index	Poor	1.07(1.02, 1.13)		**1.09(1.04, 1.15)**
	Middle	1.04(0.98, 1.09)		1.04(0.98, 1.10)
	Rich	1		1
Distance to health facility	Big problem	1.04(0.99, 1.08)		**1.08(1.03, 1.13)**
	Not big problem	1		1
Household media exposure	No	1.07(1.02, 1.12)		**1.12(1.05, 1.16)**
	Yes	1		1
**Community level variables**				
Place of residence	Urban		1	1
	Rural		1.16(1.12, 1.20)	0.95(0.90, 1.01)
Community level women illiteracy	Low		1.05(1.00, 1.11)	1.02(0.97, 1.08)
	High		1	1
Community level poverty	Low		1.04(0.99, 1.09)	0.97(0.92, 1.03)
	High		1	1
Community level media exposure	Low		1.08(1.03, 1.14)	1.03(0.98, 1.09)
	High		1	1
Community level ANC utilization	Low		1.11(1.05, 1.16)	1.03(0.99, 1.09)
	High		1	1
Country category	West SSA		1	1
	East SSA		0.64(0.52, 1.56)	0.55(0.61, 1.69)
	Central SSA		1.77(1.68, 1.86)	**1.60(1.49, 1.72)**
	South SSA		0.47(0.44, 0.50)	0.44(0.41, 0.45)

### Association of individual and community-level determinants and pentavalent vaccination dropouts among under-five children in the sub-Saharan African countries

In multivariable multilevel mixed-effect logistic regression analysis, where both the individual and community level factors were fitted simultaneously, maternal age, maternal education, place of delivery, number of ANC visits, Postnatal checkup within 2 months, household wealth status, distance to a health facility, household media exposure, and region (central sub-Saharan Africa) were significantly associated with pentavalent vaccination dropout at a p-value of < 0.05.

The odds of pentavalent vaccination dropout rates were 1.61 times higher among under-five children whose mothers ages were 15 to 24 years compared to under-five children whose mothers ages were 35 to 49 years (AOR = 1.61, 95 % CI: 1.51, 1.72). Pentavalent vaccination dropout rates were 1.30 times higher among under-five children whose mothers did not have formal education as compared to under-five children delivered from mothers with secondary and higher education levels (AOR = 1.30, 95 % CI: 1.22, 1.40). The odds of Pentavalent vaccination dropout rates were 1.65 times higher among under-five children who delivered at home as compared to children whose delivery place was a health facility (AOR = 1.65, 95 % CI: 1.57, 1.73). Pentavalent vaccination dropout was 1.34 times higher among under-five children whose mothers had four or more ANC follow-ups as compared to under-five children from mothers who had less than four ANC follow-ups during pregnancy (AOR = 1.34, 95 % CI: 1.23, 1.45).

The odds of Pentavalent vaccination dropout rates were 1.19 times higher among women in under-five children who had a postnatal checkup within 2 months as compared to under-five children who had not had a postnatal checkup within 2 months (AOR = 1.19, 95 % CI: 1.14, 1.25). Under-five children from poor household wealth status had 1.09 times higher odds of pentavalent vaccination dropout rates as compared to under-five children from the richest household wealth status (AOR = 1.09, 95 % CI: 1.04, 1.15). The odds of pentavalent vaccination dropout rates were 1.08 times higher among under-five children where distance to a health facility is a big problem compared to under-five children whose distance to a health facility is not a big problem (AOR = 1.08, 95 % CI: 1.03, 1.13). Under-five children whose family had no household media exposure had 1.12 times higher odds of pentavalent vaccination dropout rates as compared to under-five children whose family had media exposure (AOR = 1.12, 95 % CI: 1.04, 1.15). Under-five children from central sub-Saharan African countries had 1.60 times higher odds of pentavalent vaccination dropout rates compared to children from western sub-Saharan African countries (AOR = 1.60, 95 % CI: 1.49, 1.72).

## Discussion

Despite the efforts made by the governments of each country, the WHO, and other organizations that support child health during the past 20 years to promote immunization, the issue of vaccine dropout continues to be a serious concern in low and middle-income countries, such as sub-Saharan African countries.

In this study, the prevalence of pentavalent vaccination dropout among under-five children in sub-Saharan African countries was found to be 20.9 % (95 % CI: 20.64, 21.16). The finding is higher than the studies conducted in Gambia, which is 4.3 % [28], West Africa, 16.3 % [29], and Ethiopia, 17 % [30]. The potential explanation for these discrepancies may be due to differences in aggregate data and individual data. We used appended or aggregated data from individual countries that is averaged by geographic area and year. So the aggregated data results may show the highest results of pentavalent vaccination dropout rates compared to the individual data in Gambia, West Africa, and Ethiopia. On the other hand, the prevalence of pentavalent vaccination dropout rate in this study was lower than the findings conducted in Nepal, which is 28.35 % [31]. The lower prevalence of pentavalent vaccination dropout rate in this study than previous findings in Nepal could be due to differences in socio-economic status and variability in health infrastructure and health system policy, women’s attitudes towards child vaccination, and cultural differences across countries.

In the multivariable multilevel logistic regression analysis, maternal age, maternal education, place of delivery, number of ANC visits, Postnatal checkup within 2 months, household wealth status, distance to a health facility, household media exposure, and region (central sub-Saharan Africa) were significantly associated with pentavalent vaccination dropout rate at a p-value of < 0.05. In this study, the odds of pentavalent vaccination dropout rates were 1.61 times higher among under-five children whose mother’s ages were 15 to 24 years compared to under-five children whose mother’s ages were 35 to 49 years. This study's findings are supported by the previous findings in [32], [33]. The possible explanation might be that young women in the lower age extremities have less knowledge about the importance of childhood vaccination due to having less exposure to immunization information during and after pregnancy [34]. Education level was found to have a significant effect on pentavalent vaccination dropout among under-five children. Pentavalent vaccination dropout rates were 1.30 times higher among under-five children whose mothers did not have formal education as compared to under-five children born from mothers with secondary and higher education levels. This was consistent with studies reported in [20], [31], [35]. This may be due to the fact that women with higher educational levels have probably received some kind of information that covers topics like when to get vaccination for their child and may have a better awareness of the negative impact of incomplete vaccination; therefore, higher maternal education indicates greater child health knowledge [36].

The odds of pentavalent vaccination dropout rates were 1.65 times higher among under-five children who delivered at home as compared to children whose delivery place was a health facility. This finding is consistent with previous findings [5], [37], [38]. The possible explanation for the association might be that mothers who gave birth at home would have poor information about vaccination schedules for their children. Additionally, during institutional delivery, women receive education on child health care, including the advantages and schedules of immunizations, and children receive the BCG and polio zero vaccines, which serve as a starting point for successive vaccinations. Pentavalent vaccination dropout was 1.34 times higher among under-five children whose mothers had four or more ANC follow-ups as compared to under-five children from mothers who had less than four ANC follow-ups during pregnancy. It is supported by previous studies conducted in [39], [40], [41]. It is because mothers who underuse ANC services miss the opportunity to learn about the advantages and vaccination schedule. In addition, mothers who choose not to use ANC services or give birth in hospitals may place less or no value on childhood immunization compared to optimal ANC users who come from the same socioeconomic status, and they may miss out on postnatal counselling about child immunization. This is another likely explanation for the pentavalent vaccine dropout [42], [43].

Children with no a history of postnatal checkup within 2 months had 1.19 times higher pentavalent vaccination dropout rates as compared to under-five children who had a postnatal checkup within 2 months. It is consistent with studies reported in [44], [45], [46], [47]. This might be plausible since postnatal care for the full immunization of children can be explained by the fact that mothers have more opportunities to be exposed to messages about the advantages of immunization for children, which motivates them to get their children fully immunized. Postnatal care visits foster trust and communication between mothers and healthcare providers, which may influence mothers' health service-seeking behaviors that are associated with child vaccination [47], [48].

Under-five children from poor household wealth status had 1.09 times higher odds of pentavalent vaccination dropout rates as compared to under-five children from the richest household wealth status. It is in line with study findings in [49], [50], [51], [52]. This may be due to the fact that children from low-income households have inadequate access to healthcare, poor health care practices, and poor health-seeking behaviour. As a result, the child has a higher probability of vaccination dropout [5]. The study at hand also revealed that pentavalent vaccination dropout rates were 1.08 times higher among under-five children where distance to a health facility is a big problem compared to under-five children whose distance to a health facility is not a big problem. This is supported by the previous studies conducted in [19], [53], [54]. The possible explanation might be that if the distance to a health facility is considered a big problem, women are less likely to utilise health services such as antenatal and postnatal care due to poor access to transportation services to the health facility, and consequently, they would not acquire health care services such as vaccination services.

Under-five children whose family had no household media exposure had 1.12 times higher odds of pentavalent vaccination dropout rates as compared to under-five children whose family had media exposure. This is consistent with the studies conducted in [19], [42], [55]. The possible explanation could be that women who got messages through radio, television, and newspapers about family health care services had a higher chance of avoiding pentavalent vaccination dropout rates. Thus, the likelihood of having good awareness of childhood vaccination is higher among those who were exposed than among those who did not get a chance to be exposed to such media. Geographical region was significantly associated with pentavalent vaccination dropout among under-five children in Sub-Saharan African countries. Under-five children from central sub-Saharan African countries had 1.60 times higher odds of pentavalent vaccination dropout rates compared to children from western sub-Saharan African countries. This might be related to the difference in the availability of health facilities. In particular, in western sub-Saharan Africa, where the quality of health care services is better than in central sub-Saharan Africa, women from western sub-Saharan Africa are more informed about childhood vaccination due to economic and technological advancements [56].

## Strength and limitations of the study

The study's strength was the utilization of recently conducted large-sample national demography and health surveys from 33 sub-Saharan African countries. The representativeness of our finding, however, may have been impacted by the fact that some sub-Saharan African countries have not carried out a demographic and health survey since 2010. Furthermore, the study was limited in its ability to include other variables that might have been associated with the outcome variables due to a lack of some important variables in the DHS dataset, such as maternal psychological factors, behavioral issues such as vaccine hesitancy, and social determinants such as disasters.

## Conclusions and recommendation

This study concludes that pentavalent vaccination dropout rates in sub-Saharan Africa countries among under-five children were high. The study identified that both individual and community-level variables were determinants of pentavalent vaccination dropout rates. Therefore, the Government and ministry of health in Sub-Saharan Africa countries should give attention to those mothers of under-five children who reported distance as a big problem to in accessing health facilities and for women who do not utilize antenatal and postnatal check-ups while designing policies and strategies targeting reducing pentavalent vaccination dropout rates in sub-Saharan Africa.

## Ethical approval and consent to participate

This study is a secondary analysis of the DHS data, so it does not require ethical approval. For conducting our study, we registered and requested the dataset from DHS online archive and received approval to access and download the data files. According to the DHS report, all participant data were anonymized during the collection of the survey data. More details regarding DHS data and ethical standards are available online at https://www.dhsprogram.com.

## CRediT authorship contribution statement

**Alebachew Ferede Zegeye:** Conceptualization, Data curation, Formal analysis, Funding acquisition, Investigation, Project administration, Resources, Software, Validation, Writing – original draft, Writing – review & editing. **Chilot Kassa Mekonnen:** Formal analysis, Funding acquisition, Investigation, Methodology, Writing – original draft. **Hailemichael Kindie:** Data curation, Methodology, Writing – original draft, Writing – review & editing. **Belayneh Shetie Workneh:** Conceptualization, Data curation, Methodology, Writing – original draft. **Desale Bihonegn Asmamaw:** Formal analysis, Methodology, Software, Writing – review & editing. **Tadesse Tarik Tamir:** Formal analysis, Methodology, Supervision, Visualization, Writing – original draft, Writing – review & editing.

## Declaration of competing interest

The authors declare that they have no known competing financial interests or personal relationships that could have appeared to influence the work reported in this paper.

## Data Availability

Data will be made available on request.
